# TiO_2_ Nanosphere/MoSe_2_ Nanosheet-Based Heterojunction Gas Sensor for High-Sensitivity Sulfur Dioxide Detection

**DOI:** 10.3390/nano15010025

**Published:** 2024-12-27

**Authors:** Lanjuan Zhou, Chang Niu, Tian Wang, Hao Zhang, Gongao Jiao, Dongzhi Zhang

**Affiliations:** State Key Laboratory of Chemical Safety, College of Control Science and Engineering, China University of Petroleum (East China), Qingdao 266580, China; zhlj@upc.edu.cn (L.Z.); niuchang123456789@163.com (C.N.); wangtian_2020@163.com (T.W.); zhshy2020zhh@163.com (H.Z.); jiaogongao@163.com (G.J.)

**Keywords:** SO_2_ sensor, hydrothermal method, TiO_2_/MoSe_2_ composites, heterojunction

## Abstract

With the growing severity of air pollution, monitoring harmful gases that pose risks to both human health and the ecological environment has become a focal point of research. Titanium dioxide (TiO_2_) demonstrates significant potential for application in SO_2_ gas detection. However, the performance of pure TiO_2_ is limited. In this study, TiO_2_ nanospheres and MoSe_2_ nanosheets were synthesized using a hydrothermal method, and the gas-sensing properties of TiO_2_/MoSe_2_ nanostructures for SO_2_ detection were investigated. The TiO_2_/MoSe_2_ composites (with a TiO_2_-to-MoSe_2_ volume ratio of 2:1) were characterized by scanning electron microscopy (SEM), transmission electron microscopy (TEM), X-ray photoelectron spectroscopy (XPS), and X-ray diffraction (XRD). The TiO_2_/MoSe_2_ sensor exhibited high sensitivity to SO_2_; the response to 100 ppm of SO_2_ reached as high as 59.3, with a significantly shorter response and recovery time (15 s/13 s), as well as excellent repeatability, selectivity, and long-term stability. The experimental results suggest that the enhanced SO_2_ adsorption capacity of the TiO_2_/MoSe_2_ composite can be attributed to the formation of an n-n heterojunction and the unique microstructural features of TiO_2_/MoSe_2_. Therefore, the TiO_2_/MoSe_2_ sensor represents a promising candidate for rapid SO_2_ detection, providing a theoretical foundation for the development and application of high-performance SO_2_ sensors.

## 1. Introduction

Air pollution, particularly from toxic gases, poses a significant threat to both ecological systems and human health [1,2]. As a major atmospheric pollutant, sulfur dioxide (SO_2_) is classified as toxic by the World Health Organization (WHO) [3,4,5]. It is primarily emitted from the burning of fossil fuels in power plants, industrial activities, and natural sources like volcanic eruptions and forest fires [6,7,8]. Long-term exposure to high SO_2_ concentrations can irritate the eyes, respiratory system, and skin, leading to conditions such as asthma, bronchitis, and even lung failure [9,10]. Additionally, SO_2_ contributes to acid rain formation, further damaging ecosystems [11,12]. Consequently, there is an urgent need for high-sensitivity, low-cost SO_2_ sensors to monitor and mitigate its environmental and health impacts. However, unlike many other gases, SO_2_ molecules exhibit strong interactions with the surface of sensing materials, which may result in slow adsorption and desorption processes. Additionally, SO_2_ can react with metal oxide surfaces to form sulfuric acid or sulfite species, leading to changes in the surface morphology and chemical state of the material, which can impact the stability and long-term performance of the sensor. Moreover, due to the low concentration of SO_2_ in ambient air (typically in the ppb range), sensors are required to have high sensitivity. The rapid and reversible adsorption of SO_2_ necessitates materials with optimized electronic properties and fast charge transport mechanisms to ensure quick response and recovery times. These challenges highlight the need for the development of novel materials to enhance the performance of SO_2_ sensors.

In recent years, metal–semiconductor oxide (MOS) gas sensors have found widespread application in detecting toxic, harmful, flammable, and explosive gases due to their simple structure, ease of preparation, low cost, high practicality, and excellent sensing performance [13,14,15]. Among these, binary metal oxides are characterized by relatively simple and stable crystal structures, and their surfaces often exhibit chemical activity, which facilitates effective interaction with gas molecules [16,17,18]. Titanium dioxide (TiO_2_), a typical binary metal oxide, is an n-type semiconductor with a work function of 4.3 eV. It is extensively used in various gas detection applications because of its simple crystal structure, excellent stability, wide bandgap, surface chemical activity, and redox properties [19]. Shooshtari et al. synthesized a titanium dioxide nanowire-based ethanol gas sensor and conducted an in-depth study on the effects of temperature and humidity, two key factors, on the sensor’s performance at four different temperatures and under various morphologies. They discussed the optimal growth and testing conditions for gas sensing to minimize the impact of humidity and temperature [20]. Zeng et al. developed a room-temperature SO_2_ gas sensor based on TiO_2_/rGO, providing a novel approach for miniaturized, integrated, and high-performance SO_2_ gas sensors. However, the sensor exhibits a response of only 3.46% to 20 ppm SO_2_, which is relatively moderate, and the response recovery time is excessively long, reaching 456 s/134 s [21]. Thangamani et al. reported that a PVF/TiO_2_ nanofilm gas sensor exhibited a high response of 83.75% to 600 ppm SO_2_ at 150 °C, along with excellent selectivity and outstanding long-term stability; however, the response recovery time is similarly excessive, reaching 66 s/107 s [22]. The microstructure and morphology of nanomaterials can be optimized and tailored through effective methods such as hydrothermal, electrospinning, and sol–gel techniques, which significantly enhance the gas-sensitive properties of these materials [23]. Among these techniques, TiO_2_ nanomaterials synthesized via the hydrothermal method are particularly effective for SO_2_ detection [24].

To enhance the response, recovery, and reversibility of MOS-based SO_2_ sensors, various strategies have been developed, including doping MOS nanomaterials with other sensing materials to form heterostructures, such as 2D materials and graphene [25]. MoSe_2_, a 2D transition metal disulfide, has garnered attention due to its atomic thickness, which increases its surface area and contact with gas molecules, enhancing sensitivity. Additionally, MoSe_2_ possesses a band gap of 1.52 eV, high electron mobility, good electrical conductivity, and effective adsorption/desorption properties, making it a popular choice for gas sensors [26]. For instance, Pan et al. synthesized MoSe_2_-decorated α-FeO nanocomposites via a hydrothermal method, where the formation of an n-n heterojunction between MoSe_2_ and α-FeO hollow nanospheres significantly enhanced H_2_S sensing performance [27]. Similarly, Liu et al. prepared CuO/MoSe_2_ nanocomposites, achieving up to 20% response to 20 ppm H_2_S, showing potential for high-performance H_2_S sensors [28]. To address the issues of low sensitivity and long response recovery time in traditional SO_2_ sensors, we propose a highly sensitive TiO_2_/MoSe_2_ composite material sensor with fast response and recovery.

In this study, TiO_2_/MoSe_2_ composites were successfully synthesized using a simple hydrothermal method for SO_2_ sensing applications. The TiO_2_/MoSe_2_ sensor exhibited significantly improved response compared to single-material sensors. Additionally, the composite demonstrated fast response, strong selectivity, and long-term stability. The sensing mechanism of the TiO_2_/MoSe_2_ composite was further explored, focusing on the n-n heterojunction and the active sites on the material’s surface.

## 2. Experiments

### 2.1. Materials

Titanium sulfate (Ti(SO_4_)_2_, 99.9%), urea (CO(NH_2_)_2_, 99%), and sodium borohydride (NaBH_4_, 96%) were from Sinopharm Chemical Reagent Co., Ltd. (Shanghai, China). Sodium molybdate dihydrate (Na_2_MoO_4_·2H_2_O, 99%) and selenium powder (Se, 99.9%) was from Macklin Biochemical Co., Ltd. (Shanghai, China). Ethanol (C_2_H_6_O, 99.7%) was from Titan Scientific Co., Ltd. (Shanghai, China). Hydrazine hydrate (N_2_H_4_·H_2_O, 80%) was from Beilian Fine Chemicals Development Co., Ltd. (Tianjin, China).

### 2.2. Sensor Fabrication

First, 12 g of Ti(SO_4_)_2_ was weighed and dissolved with 6 g of urea in 100 mL of deionized water. After stirring for 1.5 h and ultrasonic treatment for 0.5 h, a uniformly dispersed solution was obtained. After that, the solution was sealed in a reactor and kept at 180 °C for 3 h. After heating and cooling, a TiO_2_ solution without impurities was obtained by centrifugal washing with deionized water several times. Finally, the solution was dried at 60 °C to obtain a high-purity TiO_2_ powder.

**Figure 1:**
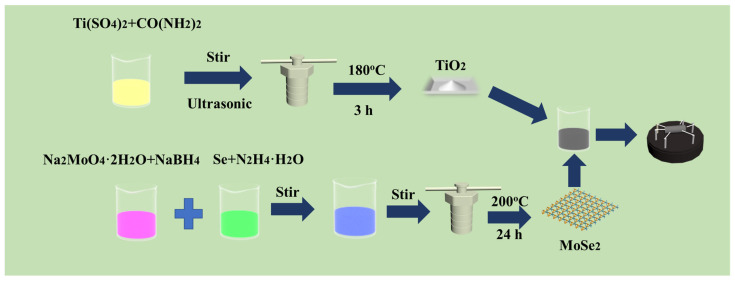
Synthesis process of TiO_2_/MoSe_2_ composite sensor.

For the preparation of MoSe_2_, 0.1 g of NaBH_4_ and 0.6 g of Na_2_MoO_4_·2H_2_O were weighed, dissolved in 100 mL of deionized water, and then stirred. At the same time, 0.493 g of Se powder and 10 mL of hydrazine hydrate were evenly mixed, then continuously injected into the mixed solution prepared in the first step, stirred for 1 h, and underwent ultrasonic treatment for 1 h. The blended solution was kept at 200 °C for 48 h. After cooling, the solution was alternately centrifuged with deionized water and ethanol, and the obtained solution was dried in a vacuum environment. The most important step is to place the dried powder in a 700 °C calcination furnace under the protection of Ar gas for 2 h to obtain the required MoSe_2_. Figure 1 describes the preparation process of the TiO_2_ and MoSe_2_ nanomaterials and the preparation of the TiO_2_/MoSe_2_ sensors.

During the experiment, the laboratory temperature is maintained at 25 °C with a relative humidity of 45% RH. Because humidity affects the conductivity of materials, typically causing a change in electrical resistance when moisture is absorbed, the electrical conductivity decreases as the moisture content increases. In a dry environment, as moisture decreases, the resistance increases. The sensor is placed in a sealed chamber of the automatic gas mixing system, where both temperature and humidity are kept constant, matching the environmental conditions of the laboratory. Unless otherwise specified, the temperature is maintained at 25 °C and the humidity at 45% RH. To detect sulfur dioxide (SO_2_) gas concentrations ranging from 0.5 to 100 ppm, the corresponding SO_2_ concentration is pre-calculated and injected into the first gas chamber. After measuring the data at the specified SO_2_ concentration, the automatic gas mixing system introduces argon gas into the first gas chamber to purge any residual SO_2_ gas, allowing the sensor to recover. Each exposure and recovery period lasts for 100 s. The sensor response, denoted as S, is defined as the ratio of Ra to Rg, where Ra is the resistance of the sensor in air and Rg is the resistance in SO_2_ gas.

## 3. Results and Discussion

### 3.1. Characterization

The crystal structures of TiO_2_, MoSe_2_, and TiO_2_/MoSe_2_ were characterized using transmission electron microscopy (TEM) (JEOL JEM-2100, Beijing, China), with the lattice spacing of TiO_2_/MoSe_2_ determined by high-resolution TEM. The surface morphology of the sensor material was examined using a scanning electron microscope (SEM, Hitachi S-4800, Beijing, China). The internal structures of the materials were analyzed by XRD (Rigaku D/Max-2550, Beijing, China) with Cu Kα radiation (k = 0.15418 nm). The chemical composition was determined using an Al-Kα X-ray excitation source and XPS characterization with thermal science instruments.

**Figure 2:**
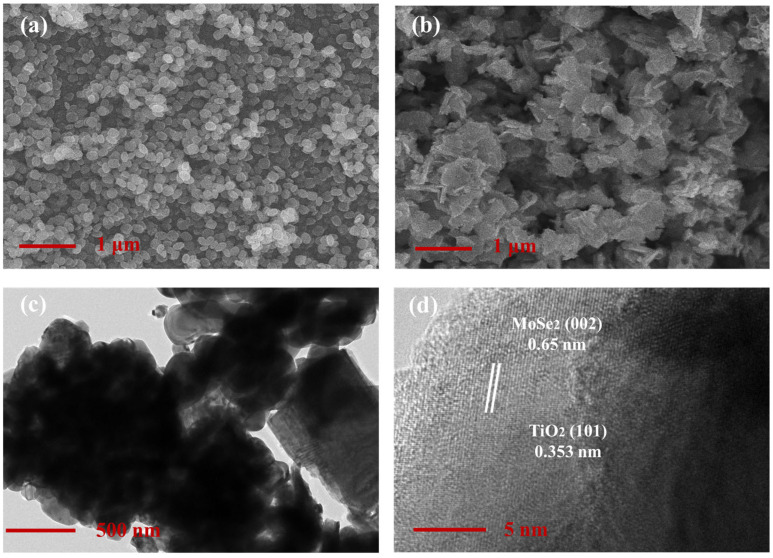
SEM images of (**a**) TiO_2_ nanocomposite and (**b**) MoSe_2_ nanocomposite; (**c**) TEM images of TiO_2_/MoSe_2_ nanocomposite; (**d**) HRTEM images of TiO_2_/MoSe_2_ nanocomposite.

The microscopic morphology of TiO_2_ and MoSe_2_ prepared by SEM was studied. Figure 2c show the TEM images of the TiO_2_/MoSe_2_ composite. TiO_2_ nanoparticles are attached to the surface of MoSe_2_ nanosheets and are in good contact with each other without any aggregation, indicating the successful preparation of the TiO_2_/MoSe_2_ composite. The SEM image of MoSe_2_ is shown in Figure 2b, where the MoSe_2_ exhibits irregular and fragmented sheet-like structures due to ultrasonic treatment. Figure 2a shows SEM images of TiO_2_ nanoparticles, confirming the successful preparation of the TiO_2_ material. The HRTEM image of the TiO_2_/MoSe_2_ composite in Figure 2d reveals lattice fringes. From the image, the TiO_2_ (101) plane corresponds to a lattice spacing of 0.353 nm, while the MoSe_2_ lattice spacing is 0.65 nm, corresponding to the (002) plane [29].

**Figure 3:**
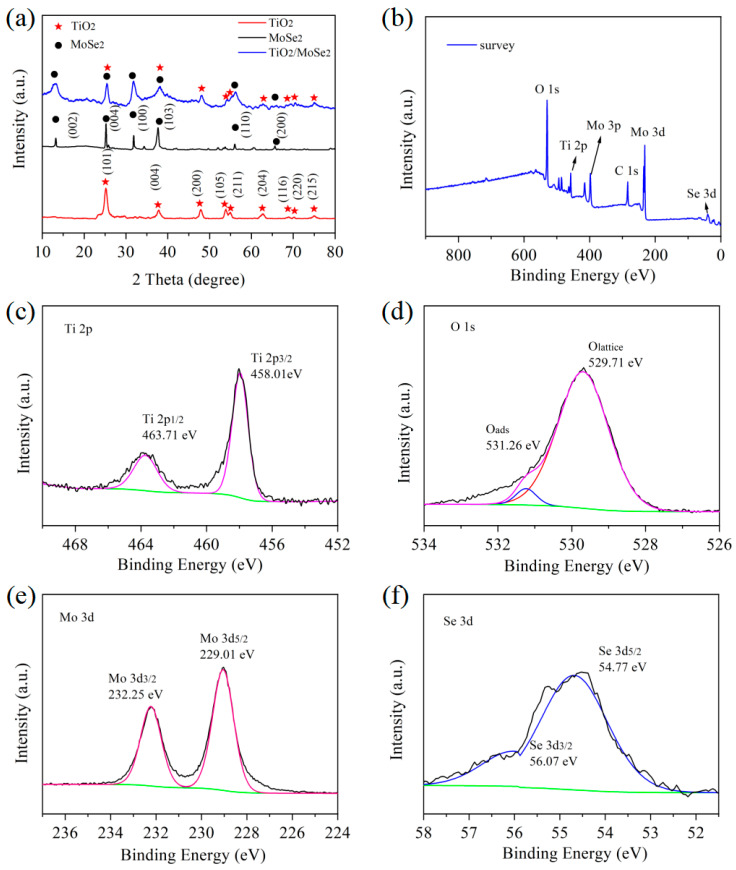
(**a**) XRD patterns of TiO_2_/MoSe_2_, TiO_2_, and MoSe_2_ nanocomposites; XPS spectra of TiO_2_/MoSe_2_ nanocomposite: (**b**) survey spectrum, (**c**) Ti 2p, (**d**) O 1s, (**e**) Mo 4d, and (**f**) Se 3d.

The crystal surfaces of TiO_2_, MoSe_2_, and TiO_2_/MoSe_2_ nanomaterials were characterized by XRD. Figure 3a describes the XRD patterns of the TiO_2_, MoSe_2_, and TiO_2_/MoSe_2_ nanomaterials in the scanning range of 10–70°. In the XRD pattern of TiO_2_, characteristic peaks appeared at 25.26°, 37.65°, 47.95°, 53.74°, 54.87°, 62.91°, 68.85°, 70.14°, and 74.96°, which were indexed to (101), (004), (200), (105), (211), (204), (116), (220), and (215), which is coincident with the standard card (JCPDS card No. 89-4921) [30]. The diffraction peaks of MoSe_2_ nanomaterials at 2θ are 13.20°, 25.26°, 31.85°, 37.65°, 55.99°, and 65.65°, corresponding to the (002), (004), (100), (103), (110), and (200) crystal planes, respectively. The obtained XRD pattern conforms to the standard card (JPCDS card No. 29-0914) [31]. The XRD patterns of the TiO_2_/MoSe_2_ composites show the diffraction peaks of the MoSe_2_ and TiO_2_ nanomaterials, which indicates the successful synthesis of TiO_2_/MoSe_2_ composites. In addition, there are no additional diffraction peaks observed in the XRD pattern, which indicates that there are no impurities in the prepared composite samples.

The elemental composition and valence states of the TiO_2_/MoSe_2_ composite samples were further determined by XPS. For the XPS data analysis of the TiO_2_/MoSe_2_ composite material, we performed the fitting using version 5.9 of the Avantage software, employing Gaussian–Lorentzian line shape fitting and Shirley background subtraction methods, as shown in Figure 3b–f. Figure 3b shows the total spectrum of TiO_2_/MoSe_2_ composites, indicating that there are Ti, O, Mo, and Se elements in the composites. Figure 3c shows the XPS spectrum of Ti 2p. The characteristic peaks at 458.01 eV and 463.71 eV are caused by Ti 2p_3/2_ and Ti 2p_1/2_ in TiO_2_. For the XPS spectrum of O 1s (Figure 3d), the characteristic peak at 531.26 eV is attributed to the adsorbed oxygen (O_ads_) formed on the sensitive material’s surface, while the characteristic peak at 529.71 eV is caused by lattice oxygen (O_lattice_) in TiO_2_ [30]. Figure 3e shows the XPS spectrum of Mo 3d, whose characteristic peaks can be observed at 229.01 eV and 232.25 eV due to the presence of Mo 3d_3/2_ and Mo 3d_5/2_ in Mo^4+^. In Figure 3f, there are two significant characteristic peaks in the XPS spectrum of Se 3d. The characteristic peak at 54.77 eV corresponds to Se 3d_5/2_, while the characteristic peak at 56.07 eV corresponds to Se 3d_3/2_ [32].

### 3.2. SO_2_ Sensing Properties

**Figure 4:**
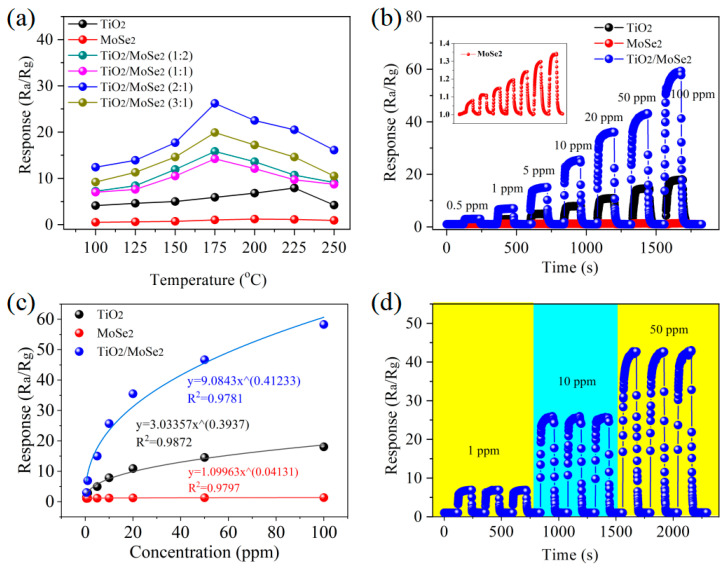
(**a**) The responses of the TiO_2_/MoSe_2_ sensor with different volume ratios to 10 ppm SO_2_ at various temperatures; (**b**) SO_2_ gas-sensing response toward different concentrations; (**c**) fitting curves of TiO_2_, MoSe_2_, and TiO_2_/MoSe_2_ sensors with different SO_2_ concentrations; (**d**) repeatability of the TiO_2_/MoSe_2_ sensor.

Gas sensors with diverse volume-doping proportions differ greatly at disparate test temperatures. Several groups of experiments are conducted to study the performances of each sensor. First, TiO_2_, MoSe_2_, and TiO_2_/MoSe_2_ (1:2, 1:1, 2:1, and 3:1) composites sensors with different volume-doping ratios were prepared. When the test environment is selected as 10 ppm SO_2_, it can be found that the TiO_2_/MoSe_2_ (2:1) sensor has the highest response. Meanwhile, the operating temperature also exerts a significant influence on the performance of the sensor. With the increase in the test temperature, the response of the sensor experiences an increase followed by a decrease. The TiO_2_/MoSe_2_ (2:1) sensor has the highest response at each working temperature, so the sensor with this doping ratio is selected for the study of gas-sensitive performance. The optimal working temperature is 175 °C, as shown in Figure 4a. The TiO_2_/MoSe_2_ (2:1) composite exhibits optimal response at 175 °C, primarily attributed to the synergistic interaction between the two materials. TiO_2_, as a wide-bandgap semiconductor, possesses excellent charge separation characteristics, while MoSe_2_, with its high electronic conductivity, facilitates efficient charge transfer. The interaction between TiO_2_ and MoSe_2_ effectively reduces charge recombination, thereby enhancing the sensor performance. Moreover, the combination of TiO_2_ and MoSe_2_ increases the specific surface area of the composite, exposing more active sites and enhancing the interaction with gas molecules. Additionally, MoSe_2_ acts as a catalyst, promoting gas reactions, while TiO_2_ provides a stable matrix, ensuring the long-term stability of the material. The electronic properties of the composite are also optimized, forming a favorable heterojunction that further improves the gas-sensing performance. Figure 4b reveals the response of the TiO_2_, MoSe_2_, and TiO_2_/MoSe_2_ sensors in SO_2_ gas with different concentrations. The response of the TiO_2_/MoSe_2_ sensor is drastically higher than that for the TiO_2_ and MoSe_2_ sensors. The TiO_2_/MoSe_2_ sensor responds up to 59 to 100 ppm SO_2_, which is 45 times better than the MoSe_2_ sensor. It can also be observed in Figure 4b that the MoSe_2_ material sensor, although exhibiting different responses in various concentrations of SO_2_ gas, shows only minor changes in the response values, with no significant differences. Figure 4c illustrates the fitting relationship between the response value (y) of the TiO_2_, MoSe_2_, and TiO_2_/MoSe_2_ sensors and the gas concentration (x), corresponding to y = 3.03357x^0.3937^, y = 1.09963x^0.04131^, and y = 9.0843x^0.41233^, respectively. Among them, the fitting coefficient of the TiO_2_/MoSe_2_ sensor is 0.9781, indicating that the curve has a high degree of fitting. Moreover, the response of the TiO_2_/MoSe_2_ sensor in 1, 10, and 50 ppm SO_2_ gas environments were tested. As shown in Figure 4d, the numerical response does not change much in the three repeated cycles, and the error remained within 1–2%, showing that the sensor has good repetition performance. The detection limit (dl) of the TiO_2_/MoSe_2_ sensor is evaluated using the formula 3[SO_2_]/((R − R_0_)/σ) [33], where σ represents the fluctuation in the electrical signal. The detection limit of the TiO_2_/MoSe_2_ sensor is calculated to be 0.25 ppm.

**Figure 5:**
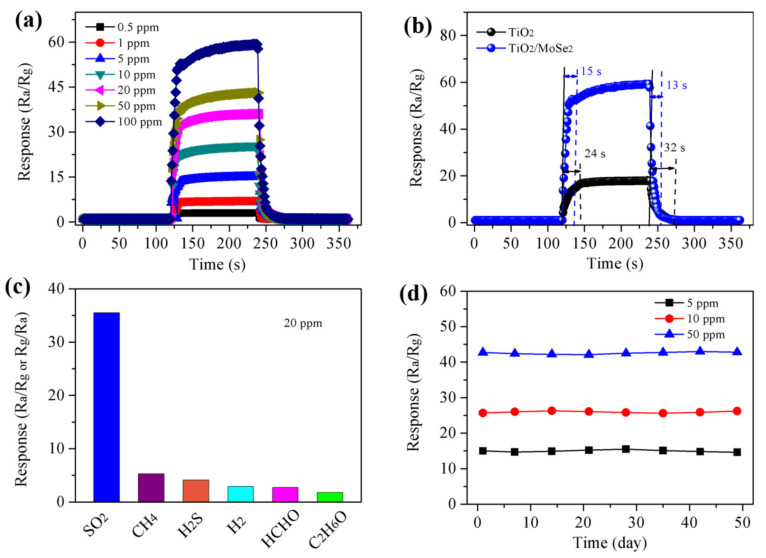
(**a**) Real-time response of TiO_2_/MoSe_2_ sensor upon 0.5–100 ppm SO_2_; (**b**) response and recovery time of TiO_2_ and TiO_2_/MoSe_2_ sensors to 100 ppm SO_2_; (**c**) selectivity and (**d**) stability of the TiO_2_/MoSe_2_ sensor.

The response/recovery characteristics are an important indicator in weighing the usefulness of gas sensors. The gas sensor underwent switching between air and gas environments with different concentrations of SO_2_ to calibrate its recovery and response ability. As shown in Figure 5a, the TiO_2_/MoSe_2_ sensor was placed in 0.5, 1, 5, 10, 20, 50, and 100 ppm SO_2_ to test its response/recovery characteristics. From this, one can infer that the TiO_2_/MoSe_2_ sensor can basically recover to its initial resistance value in an air environment. And, the sensor has an obvious response gap in different concentrations of SO_2_, showing that the sensor has excellent response/recovery characteristics. Figure 5b shows the response/recovery time of the gas sensor. The fast response of the sensor can effectively avoid the harm of toxic and harmful gases. The TiO_2_/MoSe_2_ sensor has a response time of 15 s and a recovery time of 13 s for 100 ppm SO_2_. Compared to 24 s/32 s for the TiO_2_ sensor, the response time and recovery time for the composite sensor is reduced. The results show that the synergy effect between two diverse materials improves the performance of the composite sensor. Figure 5c shows the response of the TiO_2_/MoSe_2_ sensor to the target gas and the interfering gas. It is tested under 20 ppm of different gas environments, such as formaldehyde (HCHO), methane (CH_4_), hydrogen (H_2_), hydrogen sulfide (H_2_S), and ethanol (C_2_H_6_O). SO_2_ is a common harmful gas typically found in industrial emissions and vehicle exhaust. In environments such as power plants, petrochemical factories, and urban areas with heavy traffic, SO_2_ often coexists with other gases, including formaldehyde (HCHO), methane (CH_4_), ethanol (C_2_H_6_O), hydrogen (H_2_), and hydrogen sulfide (H_2_S). Therefore, when investigating the selectivity of the sensor, we selected these interfering gases for comparison with SO_2_. It can be found that the response of the TiO_2_/MoSe_2_ sensor to the target gas is much higher than that of the interference gas, which is seven times the interference gas response. SO_2_ molecules possess a high electron affinity, which facilitates their ability to accept electrons when interacting with the TiO_2_/MoSe_2_ sensor surface, thereby enhancing the adsorption and activation of SO_2_. The heterojunction structure of the TiO_2_/MoSe_2_ composite promotes efficient charge transfer and provides favorable adsorption sites, leading to a significant interaction between SO_2_ and the sensor surface, resulting in a stronger sensor response. Compared to other gases, the difference in electron affinity of SO_2_ enables the sensor to exhibit higher selectivity and sensitivity toward SO_2_, while effectively resisting interference from gases such as HCHO, CH_4_, H_2_, H_2_S, and C_2_H_6_O. As shown in Figure 5d, the response of the TiO_2_/MoSe_2_ sensor to SO_2_ gas with different concentrations within 50 days was tested. The response numerical of the sensor at the same concentration showed no obvious change trend, indicating that the long-term stability of the TiO_2_/MoSe_2_ sensor was good.

Table 1 shows the performance comparison between the TiO_2_/MoSe_2_ sensor and the reported SO_2_ gas sensor, such as their preparation methods, response/recovery times, optimal operating temperatures, and response values [34,35,36,37]. The SO_2_ gas sensor based on the TiO_2_/MoSe_2_ composite has better sensing performance than other SO_2_ gas sensors.

### 3.3. Sensing Mechanism of SO_2_

**Figure 6:**
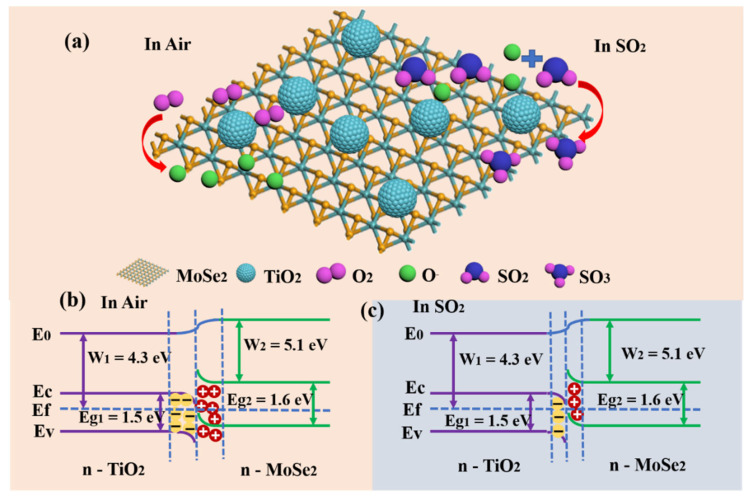
(**a**) Illustration of the TiO_2_/MoSe_2_ sensor-sensing mechanism; energy band structure of the TiO_2_/MoSe_2_ sensor (**b**) in air and (**c**) in SO_2_.

Based on the gas-sensitive property test results of the TiO_2_/MoSe_2_ sensor, it has an outstanding gas-sensitive response to SO_2_. Figure 6 shows the 3D schematic of the TiO_2_/MoSe_2_ composite sensor’s gas sensitive mechanism and energy band structure. Figure 6a shows the 3D diagram of the TiO_2_/MoSe_2_ sensor in an air environment and SO_2_ gas, which mainly describes the molecular changes in the TiO_2_/MoSe_2_ sensor in the response process. In air, O_2_ is very easily adsorbed on the surface of TiO_2_- and MoSe_2_-sensitive materials to become adsorbed oxygen. At the optimum working temperature of 175 °C, the adsorbed oxygen on the surface of sensitive materials will form O^−^. When the sensor is switched from an air environment to SO_2_, SO_2_ molecules will combine with O^−^ to produce a reaction, forming some electrons and SO_3_ gas molecules. The above reaction can be reduced to the following formula [38]:

O_2_(gas)→O_2_(ads) (1)

O_2_(ads) + 2e^−^→2O^−^ (2)

SO_2_ + O^−^→SO_3_ + e^−^ (3)

Compared with a TiO_2_ gas sensor, the performance of the TiO_2_/MoSe_2_ sensor has improved a lot. The performance improvement can be summarized as follows. First, as shown in the band structure diagram of Figure 6b,c, the n-n heterojunction will be composed between TiO_2_ and MoSe_2_. The work function of TiO_2_ is 4.3 eV and that of MoSe_2_ is 5.1 eV. When materials with different work functions contact each other, electron transfer will be generated [39]. Therefore, when the sensitive material TiO_2_ is in contact with MoSe_2_, electrons will flow from TiO_2_ with high Fermi level to MoSe_2_, so that the Fermi level reaches equilibrium, forming an electron depletion layer at TiO_2_ and an electron accumulation layer at MoSe_2_. When the TiO_2_/MoSe_2_ sensor is placed in an air environment, the adsorbed oxygen on the surface of the sensitive material can further obtain its electrons and generate O^−^ [40], which widens the electron depletion layer at the sensitive material TiO_2_, thus increasing the resistance of the sensor and leaving it in a state of high resistance. When the TiO_2_/MoSe_2_ sensor is converted from the air environment to SO_2_, SO_2_ will combine with O^−^ on the surface of the sensitive material, releasing some electrons, which will return to the sensitive material, narrowing the depletion layer and reducing the resistance of the gas sensor in this process. The existence of a heterojunction between MoSe_2_ and TiO_2_ speeds up the rate of electron transfer and shortens the response/recovery time. Moreover, the presence of a heterojunction will reduce the resistance base value of the composite sensor and cause a large change in the resistance value in the response process, thus improving the performance of the TiO_2_/MoSe_2_ sensor [41]. Second, according to the various characterizations above, TiO_2_ in the sensitive film had good contact with MoSe_2_, and TiO_2_ nanospheres were evenly scattered among the surface of MoSe_2_. The unique nanostructure of the spherical and sheet composite not only reduces the agglomeration of the sensitive material itself but also has more gas adsorption sites and diffusion channels, which improves the sensing properties of the TiO_2_/MoSe_2_ sensor towards SO_2_ gas.

## 4. Conclusions

In conclusion, micro-spheroidal TiO_2_ and MoSe_2_ nanosheets were synthesized via a hydrothermal method, and TiO_2_/MoSe_2_ nanofilms were subsequently used for SO_2_ gas sensing. The morphology of the materials was characterized by SEM, while TEM, XRD, and XPS confirmed the lattice structure, crystallinity, and elemental and valence states of TiO_2_/MoSe_2_, respectively. The TiO_2_/MoSe_2_ sensor demonstrated excellent sensitivity to SO_2_ (59.3% at 100 ppm), with fast and reversible response. Additionally, the sensor exhibited strong selectivity for SO_2_, as well as good repeatability and long-term stability. The enhanced gas-sensing performance of the composite sensor can be attributed to the formation of an n-n heterojunction; effective material contact; and the sheet-like structure, which provides numerous bonding sites for gas molecules. Therefore, the SO_2_ sensor developed in this study shows promising potential for future applications in environmental monitoring.

## Figures and Tables

**Table 1:** Performance comparison of this work with the reported SO_2_ sensors.

Materials	Method	Temp. (°C)	Res/Rec Time (s)	Response (%)	Detection Limit	Ref.
Ni-SnO_2_	Drop coating	250	52 s/45 s	5.2@10 ppm	0.1 ppm	[32]
Ag-PANI/SnO_2_	Drop coating	RT	110 s/100 s	20.1@50 ppm	0.1 ppm	[34]
SnO_2_-MoS_2_	Spin coating	RT	217 s/633 s	4.68@1 ppm	0.5 ppm	[11]
ZnO/GaN	Sputtering	RT	230 s/275 s	12.1@10 ppm	0.25 ppm	[35]
NiO-SnO_2_	Drop coating	240	25 s/35 s	10.8@100 ppm	0.5 ppm	[36]
TiO_2_-MoSe_2_	Drop coating	175	15 s/13 s	59.3@100 ppm	0.25 ppm	This work

## Data Availability

Data are contained within the article.
