# Correction: Zhou et al. TiO_2_ Nanosphere/MoSe_2_ Nanosheet-Based Heterojunction Gas Sensor for High-Sensitivity Sulfur Dioxide Detection. *Nanomaterials* 2025, *15*, 25

**DOI:** 10.3390/nano15221741

**Published:** 2025-11-19

**Authors:** Lanjuan Zhou, Chang Niu, Tian Wang, Hao Zhang, Gongao Jiao, Dongzhi Zhang

**Affiliations:** State Key Laboratory of Chemical Safety, College of Control Science and Engineering, China University of Petroleum (East China), Qingdao 266580, China; zhlj@upc.edu.cn (L.Z.); niuchang123456789@163.com (C.N.); wangtian_2020@163.com (T.W.); zhshy2020zhh@163.com (H.Z.); jiaogongao@163.com (G.J.)

In the original publication [1], the same XRD patterns were mistakenly shown for MoSe_2_ and TiO_2_/MoSe_2_ in Figure 3a and in Figures 4b and 5a. The correct Figures 3a and 4b appear below. The authors would like to apologize for any inconvenience caused and state that the scientific conclusions are unaffected.




